# A review – Prescribing iron salts: Comparison of efficacy and tolerance of different iron complexes

**DOI:** 10.4102/safp.v68i1.6260

**Published:** 2026-06-26

**Authors:** Kamilla R. Snyman, Jacques R. Snyman, Angelique Coetzee

**Affiliations:** 1 DermaV Pharmaceuticals (Pty) Ltd, Pretoria, South Africa; 2 Forte Research (Pty) Ltd, Pretoria, South Africa; 3 General Practioner, Private Practice, Pretoria, South Africa

**Keywords:** anaemia, iron deficiency anaemia, iron, iron salts, iron complex, iron supplementation

## Abstract

Iron is an essential element as it participates in a wide variety of metabolic processes, including oxygen transport, deoxyribonucleic acid synthesis and electron transport. However, as iron can form free radicals, its concentration in body tissues must be tightly regulated because, in excessive amounts, it can lead to tissue damage. Disorders of iron metabolism are among the most common diseases of humans with diverse clinical manifestations. Iron deficiency is the most common mineral deficiency, affecting approximately 20% of the population. Patients may present with an iron deficiency without anaemia, which is even more common. The World Health Organisation has estimated that approximately 8% of preschool children, 12% of pregnant women and 15% of non-pregnant women of reproductive age have anaemia, with iron deficiency being the major cause.

**Contribution:**

This review focuses on the effectiveness of different preparations to ensure more appropriate choices by prescribers that is patient-centred.

## Introduction

Iron deficiency is typically defined as a condition in which the body has depleted iron stores along with signs of compromised iron supply to tissues. Patients may present with an iron deficiency either with or without anaemia. Some functional changes may occur in the absence of anaemia, but the most functional deficits occur with the development of anaemia. In mild or moderate forms of iron deficiency anaemia (IDA), functional impairments may be present, affecting cognitive development, immune mechanisms, and work capacity. An iron deficiency during pregnancy is associated with various adverse outcomes for both mother and infant, including an increased risk of sepsis, maternal mortality, perinatal mortality, and a low birth weight.^1,2^

An iron deficiency generally occurs when iron absorption cannot keep up with metabolic demands over an extended period of time.^3^ The primary causes of iron deficiency include a low intake of bioavailable iron, increased iron requirements as a result of rapid growth, pregnancy, menstruation, excessive blood loss, infections and impaired iron absorption.^4^ The prevalence of iron deficiency increases markedly among female adolescents as a result of menstrual blood loss.^4^ Other risk factors for iron deficiency are high parity, use of chronic proton-pump inhibitors (PPIs), use of an intrauterine device, patients who have undergone bariatric surgery and vegetarian diets.^4^ A nutritional iron deficiency is a direct result of inadequate iron absorption from diet.^1,2^ Patients who consume a monotonous plant-based diet with little meat often present with low iron bioavailability.^1,2^ A triple-problem that South Africa faces is the high prevalence of human immunodeficiency virus and acquired immunodeficiency syndrome (HIV/ADIS), tuberculosis (TB) and malnutrition – creating a complex, synergistic effect that significantly increases the prevalence and severity of anaemia. HIV typically causes anaemia through chronic inflammation, direct bone marrow suppression, as well as lowering CD4 counts – ultimately reducing the production of red blood cells (RBC). HIV/AIDS weakens the immune system while impairing the body’s ability to absorb, store and utilise nutrients. Studies demonstrate that antiretroviral therapy (ART) improves haemoglobin levels regardless of the magnitude of immunosuppression and the ART initiating regimen.^5,6^ While ART improves haemoglobin (Hb) levels over time, supplementation is advised. Although IV iron works more quickly and often allows for fewer side effects, it is typically expensive, and patients are required to visit a clinic for administration. In South Africa, both these factors play an increasingly important role in patient compliance and ultimately patient outcomes.^5,6^

Patients suffering from TB are three times more likely to have severe anaemia compared to other illnesses – this is a result of infection inducing ‘anaemia of chronic disease’, where inflammatory cytokines prevent the body from utilising iron, even if iron stores are sufficient. Malnutrition in South Africa is a severe crisis – often linked to poverty along with food insecurity. Malnutrition commonly causes deficiencies in iron, folate and vitamin B12.^7,8,9,10^

### Biochemistry and physiology

Iron is an abundant element on Earth – it is a biologically essential component for living organisms.^3^ Although iron is present in geological abundance, it is often access-limited in the environment because, upon contact with oxygen, iron forms highly insoluble oxides. These oxides are thus not readily available for uptake.^3^ The human body stores iron mainly in complex forms bound to proteins (haemoprotein) as haem compounds (haemoglobin or myoglobin), haem enzymes or non-haem compounds (flavin-iron enzymes, transferrin, and ferritin).^11^

The human body needs iron for the synthesis of haemoglobin and myoglobin, as well as the formation of haem enzymes along with other iron-containing enzymes.^11^ Approximately 66% of the body’s iron is found in the haemoglobin present in circulating erythrocytes, 25% in a readily mobilisable iron store, and the last 15% is typically bound to myoglobin in the muscle tissue and in a variety of enzymes involved in the oxidative metabolism and many other cellular functions.^11^

Iron is conserved in the body by means of recycling. Iron is delivered to tissues by circulating transferrin ‒ a transporter able to capture iron released into the plasma from the intestinal enterocytes or reticuloendothelial macrophages. The binding of iron-laden transferrin to the cell-surface transferrin receptor-1 (TfR) results in endocytosis, which allows for the uptake of the metal cargo. Internalised iron is transported to the mitochondria for the synthesis of haem, as well as iron-sulphur clusters. These clusters are important parts of several metalloproteins, and excess iron is stored and detoxified in cytosolic ferritin. Once iron is absorbed, there is no physiologic mechanism for excretion of excess iron from the body other than blood loss, such as that occurring during pregnancy, menstruation or other types of bleeding.^12^

### Human requirements

Adults store around 1 g – 3 g of iron in the body, with about 1 mg of iron lost daily through the natural sloughing of dead skin cells and mucosal surfaces, and the lining of the gastrointestinal tract.^13^ Daily loss is increased to about 2 mg per day during menstruation. Iron requirements are also increased during the augmentation of body mass during neonatal and childhood growth spurts.^11,12^

### Patient diagnosis

The clinical signs and symptoms of an iron deficiency with or without anaemia are limited and often neglected ‒ the most important being fatigue, which is very nonspecific. Alterations within the epithelial cells, such as dry mouth, cheilitis, atrophic glossitis Plummer-Vinson pharyngeal webs, along with severe hair loss, are typical symptoms observed in a longstanding deficiency. In elderly patients, IDA may cause heart failure or even angina.^7,8,9,10,11,12^

It is thus crucial to confirm a diagnosis using laboratory testing. Low serum ferritin levels – which reflect exhausted stores – are the hallmark of absolute iron deficiency. Serum ferritin levels below 30 mg/L are the accepted threshold for identifying mild cases. In the presence of anaemia, ferritin levels are often lower (< 10 mg/L – 12 mg/L). In the absence of inflammation or infection, serum ferritin levels indicate the best correlation with bone marrow stainable iron (iron stored in bone marrow).^7,8,9,10,11,12^

Measuring transferrin (TF) saturation (< 16%) is unnecessary for diagnosis, although it has diagnostic value in functional deficiency when serum ferritin is unreliable. Similarly, hepcidin levels, which are low or undetectable in absolute iron deficiency, are unnecessary. Exceptions are the rare iron-refractory iron deficiency anaemia (IRIDA) patients who show low TF saturation and normal/high hepcidin and serum ferritin levels, reflecting increased macrophage iron. Measuring serum hepcidin may be diagnostic of this atypical iron deficiency, if inflammation is excluded.^7,8,9,10,11,12^

Anaemia is defined as a haemoglobin level two standard deviations below normal for age and sex.^12^

### Bioavailability

The fraction of iron absorbed from dietary uptake is typically low, ranging from 5% to 35%, depending on circumstances and the type of iron. Dietary iron occurs either as haem or non-haem. The primary sources of haem iron are haemoglobin and myoglobin from the consumption of meat, poultry and fish. Non-haem iron is obtained from cereals, pulses, legumes, fruits and vegetables. Haem iron is highly bioavailable (15% – 35%), and dietary factors have little effect on its absorption, whereas non-haem iron absorption is much lower (2% – 20%) and strongly influenced by the presence of other food components. Despite its lower bioavailability, non-haem presents in larger quantities in the diet and thus contributes more to iron nutrition than haem iron.^14,15^

The major players in influencing iron absorption are inhibitors such a phytates and calcium, and competitors, manganese, zinc and other heavy metals, while ascorbic acid and citrate facilitate absorption.^14,15^

### Dietary modification and supplementation

Dietary modifications aim to correct micronutrient deficiencies by increasing the intake of iron-rich foods – especially flesh foods, increasing consumption of fruits and vegetables rich in ascorbic acid to enhance non-haem iron absorption, and reducing the intake of tea and coffee, which may inhibit non-haem iron absorption. Iron salts, with high bioavailability, are preferred when it comes to oral iron supplementation. Iron absorption is typically higher when iron supplementation is administered on an empty stomach; however, nausea and epigastric pain may present, reducing compliance. Lower doses between meals may thus be advised or salt forms adjusted in order to reduce these side effects.^14,15^

## Key differences: Oral versus intravenous iron supplementation

### Different iron salts

Oral iron supplementation is the first-line treatment for iron deficiency. Conventional iron salts have typically been associated with a variety of side effects, which include nausea, vomiting, abdominal discomfort, constipation, diarrhoea and dyspepsia. The absorption of these salts is often reduced by ingredients in a meal, such as calcium and tannins, which oxidise the ferrous iron (Fe^2+^) to ferric iron (Fe^3+^), the latter being poorly absorbed. These limitations have resulted in the emergence of newer oral iron preparations like iron polymaltose. All iron must be reduced from the ferrous state to enter the mucosal cells.^16,17^

Iron (III)-hydroxide polymaltose complex (IPC) is classified as a stable, orally administered non-ionic Fe (III) preparation which demonstrates efficacy to correct iron deficiency anaemia. Iron (III)-hydroxide polymaltose complex exhibits low toxicity and is generally well tolerated.^16,17,18,19,20,21^

Iron (III)-hydroxide polymaltose complex is one of the few oral iron compounds that acts as a slow-release iron preparation. The polymaltose component forms a casing around the trivalent iron – this allows for slower release of the iron from the complex. Iron (III)-hydroxide polymaltose complex presents with unique advantages, including a favourable side-effect profile compared with iron (II) salts because of its slow release and its ability to be taken with meals. Iron (III)-hydroxide polymaltose complex exhibits a higher tolerability compared with iron sulphate salts. This is as a result of a reduced formation of oxygen radicals and thus decreased plasma lipid peroxidation.^16,17,18,19,20,21^

Equal amounts of iron are available from iron polymaltose complex or ferrous sulphate (FS) in correcting haemoglobin levels over a 12-week observation period. This occurs with significantly fewer gastrointestinal side effects from the former. In another study performed on anaemic pregnant rats, it was clear that IPC, ferrous fumarate (FF) and FS were all efficient in correcting IDA during pregnancy. However, in contrast to FF and FS, treatment with IPC reduced IDA-mediated oxidative stress, that is, all the analysed oxidative stress markers returned to normal levels.^18,19,20,21^

### Reviewing iron salts in South Africa

Oral iron supplementation and IV iron administration are theoretically able to effectively treat anaemia within the clinical context of South Africa. The degree of effectivity, however, largely depends on tolerance. Intravenous iron supplementation is evidently the best-tolerated option – but expensive and timely. This makes the IV iron supplement option unaffordable for most South Africans. Most patients would need to travel to a specific clinic and need to take time off work for IV iron administration, making it highly infeasible. Oral iron supplementation is thus preferred for convenience of use, availability and costs – despite adverse effects associated with consumption. The key is thus to minimise or eliminate side effects by selecting an iron salt best suited to the individual patient.

Available products in South Africa are summarised in Table 2.

**TABLE 1:** Differences between oral iron supplementation and intravenous iron supplementation.

Pharmacokinetics and/or Pharmacodynamics	Oral iron	IV iron
Onset of action	Slow (months for full correction)^14^	Rapid (days or weeks, often one visit)
Effectiveness	Often low because of poor absorption	High (bypasses GI absorption)^13^
Side effects	Gastrointestinal (nausea, constipation)^14^	Infusion reactions, arthralgia, headache
Convenience	Convenient, can be taken at home	Requires a clinic visit for infusion
Cost	Low cost^14^	Higher cost

*Source*: Monsen, Hallberg L, Layrisse M, et al. Estimation of available dietary iron. Am J Clin Nutr. 1978;31(1):134–141. https://doi.org/10.1093/ajcn/31.1.134; Das SN, Devi A, Mohanta BB, Choudhury A, Swain A, Thatoi PK. Oral versus intravenous iron therapy in iron deficiency anemia: An observational study. J Family Med Prim Care. 2020;9(7):3619–3622. https://doi.org/10.4103/jfmpc.jfmpc_559_20

Note: Please see the full reference list of Snyman KR, Snyman JR, Coetzee A. A review – Prescribing iron salts: Comparison of efficacy and tolerance of different iron complexes. S Afr Fam Pract. 2026;68(1), a6260. https://doi.org/10.4102/safp.v68i1.6260

IV, intravenous, GI, Gastrointestinal.

**TABLE 2:** Oral iron supplements available in South Africa.^16,17,18,19,20,21,22,23,24,25,26,27,28,29,30,31,32,33,34,35,36,37,38^

Type of iron per brand available in South Africa	Efficacy	Tolerance	Cost	Availability	Ideal patient population
Iron polymaltose Ferrimed^®^	Effective (high bio-availability)	Improved tolerability. Low GI effects.	3	5	Poor adherence to other Iron salts because of side effects.
Iron pyrophosphate Sucrosomial^®^ (SiderAL^®^ FORTE 15/QuadroFER^®^)	Effective (reasonable bio-availability)	Acceptable tolerability. Low rebuild of iron stores. Low GI side effects.	5	5	Poor adherence to other Iron salts because of side effects.
Ferrous bisglycinate Ferrous Forte^®^	High Efficacy (reasonable bio-availability)	Poor tolerability. GI side effects. Multiple tablets required that increase side effects.	5	5	Patients who are able to tolerate.
Ferrous fumarate Autrin^®^	High Efficacy (reasonable bio-availability)	Poor tolerability. GI side effects. Multiple tablets required that increase side effects.	3	5	Patients who are able to tolerate.
Ferrous sulphate Clicks Pay Less Iron Supplement Tablets	Effective (medium bio-availability)	Significant intolerable side effects.	1	5	Patients who are able to tolerate. Often First choice in low-income environments – requires adherence follow-up.

Note: Numbers 1-5 are the scores for the scale representation where 1 is the lowest and 5 is the highest. Please see the full reference list of Snyman KR, Snyman JR, Coetzee A. A review – Prescribing iron salts: Comparison of efficacy and tolerance of different iron complexes. S Afr Fam Pract. 2026;68(1), a6260. https://doi.org/10.4102/safp.v68i1.6260

GI, Gastrointestinal.

### Patient dosing schedule and routine of elemental iron

Dosage and treatment duration for Iron deficiency are individualised according to the extent of deficiency (see Table 3).

**TABLE 3:** An outline of the dosing schedule and routine of elemental Iron.

Condition	Administration	Dosage and therapy duration as elemental Iron (daily)	Dosage and therapy duration in pregnant women (daily)	Dosage as elemental Iron treatment in children (according to age)		
				Infants – 1 year (daily)	1–12 years (daily)	12 years (daily)
Deficiency without anaemia^12^	All doses may be taken as a single dose or in divided doses.	50 mg – 100 mg for 1–2 months.	100 mg	As drops 15 mg – 15 mg	As drops or syrup: 25 mg – 50 mg	As drops or syrup or tablets or chew tablets: 50 mg – 100 mg
Iron deficiency Anaemia^12^	All doses may be taken as a single dose or in divided doses.	100 mg – 300 mg for 3–5 months or until normalisation of Hb level is attained. Then continue with 100 mg daily for several weeks.	200 mg – 300 mg until the target Hb is achieved. Thereafter, continue with 100 mg daily at least until the end of pregnancy.	As drops 25 mg – 50 mg	As drops or syrup: 50 mg – 100 mg	As drops or syrup or tablets or chew tablets: 100 mg – 300 mg
Prophylaxis of Iron deficiency in pregnancy^12^	All doses may be taken as a single dose or in divided doses.	50 mg – 100 mg	-	-	-	-
Prevention in high-risk patients^12^	All doses may be taken as a single dose or in divided doses.	100 mg	-	-	-	-

*Source*: Lopez A, Cacoub P, Macdougall I, Peyrin-Biroulet L. Iron deficiency anaemia. Lancet. 2016;387(10021):907–916

Note: Please see the full reference list of Snyman KR, Snyman JR, Coetzee A. A review – Prescribing iron salts: Comparison of efficacy and tolerance of different iron complexes. *S Afr Fam Pract*. 2026;68(1), a6260. https://doi.org/10.4102/safp.v68i1.6260

Hb, haemoglobin.

## Iron polymaltose versus iron pyrophosphate

Iron absorption from water-soluble forms of iron is inversely proportional to the iron status in the human body. Iron pyrophosphate, also known as ferric pyrophosphate, is a yellowish-white solid, practically insoluble in water. Iron polymaltose is a brown, amorphous powder which is soluble in water. More soluble iron compounds not only exhibit better overall absorption and can be used at lower fortification levels, but they also have the added advantage that, because their absorption is upregulated in iron deficiency, they innately ‘target’ iron-deficient individuals treated as such.^28,29^

Iron pyrophosphate is typically transported within a phospholipid and sucrester matrix known as Sucrosomial^®^ Iron (SI). Sucrosomial^®^ Iron a new and innovative oral iron formulation, in which iron pyrophosphate is protected by a phospholipid bilayer plus a sucrester matrix (sucrosome), which is absorbed through paracellular and transcellular routes. Available evidence supports oral SI iron as a valid option for iron deficiency treatment.^30^

A recent study, published in 2024, demonstrated that IPC and SI were equally effective in the treatment of IDA as well as iron deficiency without anaemia (IDWA), at equal dosages of elemental iron. At the recommended doses, oral iron therapy also does not induce intestinal inflammation.^31^

### Response and follow-up

Oral iron-replacement therapy is the mainstay of treatment for iron deficiency with or without anaemia; however, it is often poorly tolerated or ineffective. Haemoglobin response at day 14 of oral iron supplementation may be useful in assessing whether and when to transition patients from oral to intravenous (IV) iron supplementation.^39^ Although IV iron supplementation is increasingly recognised as a crucial, effective intervention for managing IDA in South Africa, particularly when oral iron is poorly tolerated, ineffective, or when rapid correction is required – IV iron carries risks, including rare but severe hypersensitivity reactions as well as higher costs and the need for medical infrastructure. When comparing IV iron with oral agents – IV raises Hb levels significantly faster than oral iron and is preferred for patients who cannot tolerate oral iron, have severe deficiencies or suffer from conditions that hinder iron absorption (e.g. IBD). Intravenous iron can replete iron stores in one or a few visits, whereas oral iron often requires months of daily administration. Oral iron supplements are often accompanied by gastrointestinal side effects such as constipation, nausea and abdominal pain, where IV is generally well tolerated with fewer side effects. Although both are effective, IV is superior for speed and overcoming absorption issues, while oral iron is best for low-cost, long-term maintenance, with oral iron being less expensive and easier to obtain. Intravenous iron requires a clinical visit, medical scheme approval, and professional administration.^32,33,39^

## Conclusion

Iron deficiency may not only translate into anaemia but also affects the entire energy transport process at a mitochondrial level. The latter often responds early, with patients ‘feeling better’ even before there is a change in haemoglobin levels. Choosing an iron supplement should be guided by evidence. At present, the evidence suggests that ferrous ions in a modified form are well absorbed and result in fewer gastrointestinal side effects. It is often these side effects that jeopardise compliance and response to treatment. There are limited head-to-head studies comparing iron polymaltose to iron pyrophosphate; however, the most recent double-blind, controlled study demonstrated no difference in the response to these compounds in children with iron deficiency with or without anaemia.^14^ The choice between products should therefore be guided by cost-effectiveness as well as the amount of elemental iron provided per dose.
